# Full-Field Stimulus Threshold: A Key Functional Outcome Measure in Retinal Diseases and Clinical Trials

**DOI:** 10.3390/jcm15166492

**Published:** 2026-08-21

**Authors:** Nathan Macha, Minzhong Yu

**Affiliations:** 1 Montana College of Osteopathic Medicine, Rocky Vista University, Billings, MT 59106, USA; 2 Department of Ophthalmology and Visual Sciences, University Hospitals Eye Institute, Case Western Reserve University, Cleveland, OH 44106, USA; 3 Cole Eye Institute, Cleveland Clinic Foundation, Cleveland, OH 44106, USA; 4 Department of Ophthalmology, Cleveland Clinic Lerner College of Medicine of Case Western Reserve University, Cleveland, OH 44195, USA

**Keywords:** full-field stimulus threshold, Leber congenital amaurosis, retinitis pigmentosa, choroideremia, Stargardt disease, age-related macular degeneration, birdshot chorioretinopathy, Jalili syndrome

## Abstract

Full-field stimulus threshold (FST) testing is a psychophysical method used to assess global retinal function, particularly in patients with severe visual impairment where conventional perimetry is unreliable. This review explores the development, clinical protocols, and applications of FST in retinal diseases, with a focus on its role in inherited retinal dystrophies (IRDs) such as Leber congenital amaurosis (LCA) and retinitis pigmentosa (RP). FST has emerged as a key functional outcome measure in clinical trials, particularly in evaluating novel gene therapies for IRDs. Its fixation-independent nature and ability to detect residual visual function make it valuable for assessing disease progression and treatment efficacy. However, challenges remain regarding standardization and test variability. Ongoing efforts seek to standardize and optimize FST protocols and establish it as a standardized metric in both clinical and research settings.

## 1. Introduction

Full-field stimulus threshold (FST) testing is a psychophysical measure used to determine the lowest light intensity a subject can detect under scotopic, mesopic, or photopic conditions. During FST testing, the threshold for detecting full-field Ganzfeld flashes is measured at a specified time point, providing a global assessment of retinal sensitivity. Initially developed to assess inherited retinal dystrophies (IRDs) such as Leber congenital amaurosis (LCA), FST has evolved into an essential tool for evaluating global retinal function. It is particularly valuable for monitoring disease progression and therapeutic responses in patients with severe retinal disease, where conventional perimetry may be unreliable [1,2]. Unlike standard visual field tests, which require central fixation, FST is fixation-independent, making assessment feasible in patients with profound visual impairment or nystagmus [3,4].

FST has become an increasingly important functional outcome measure in retinal clinical trials because functional endpoints traditionally used become severely limited in populations with advanced disease. Best-corrected visual acuity (BCVA) is largely preserved until late disease stages because it reflects only foveal function, making it insensitive to slowly progressive and hetergenous loss in most IRDs. In a retrospective dominance analysis in choroideremia, BCVA ranked lowest among functional measures for detecting disease progression [5]. Additionally, a phase 3 choroideremia gene therapy trial failed its primary endpoint of ≥15-letter BCVA improvement despite signs of biological efficacy [6]. Full-field electroretinography (ffERG) becomes non-detectable above noise once photoreceptor loss is advanced. For example, many patients with retinitis pigmentosa (RP) have non-recordable full-field cone responses (<10 μV) on conventional recording, even while retaining ambulatory or reading vision, which limits its use in tracking advanced disease populations in clinical trials [7]. Finally, microperimetry provides spatially resolved sensitivity maps but can be limited by patient fatigue, response variability, and unstable fixation. Anatomic-functional correlations of microperimetry were also found to weaken longitudinally in RP [8,9].

In contrast, FST is fixation-independent and quantifies residual whole-field photoreceptor sensitivity, including surviving peripheral retina that macula-weighted tests like BCVA and microperimetry do not capture. In advanced RP with non-detectable rod ERG, FST thresholds correlated strongly with anatomical markers, including ellipsoid zone area and autofluorescent ring dimensions, and residual visual field area in advanced disease [10]. Because of these properties, FST has increasingly been grouped with optical coherence tomography (OCT) and microperimetry as a sensitive and objective measure for advanced retinal degeneration.

The translational relevance of FST has solidified in recent years. FST served as a key secondary endpoint in the pivotal phase 3 trial of voretingene neparvovec (VN) for RPE65-associated retinal dystrophy, with improvements of approximately 2 log units by year 1 that were durable through 3–4 years [11,12]. More recently, FST was an efficacy measure in the phase ½ trial of ATSN-101 for GUCY2D-associated LCA, where high-dose treatment produced mean improvements of 20 dB (approximately 2 log units), which was sustained to 18 months [13]. FST has also been incorporated into natural history studies for conditions such as CEP290-associated disease [14].

Despite expanding use of FST, prior reviews are either single-center methodological and experiential perspectives centered on LCA or scoping reviews that catalog protocol heterogeneity across studies without comparative endpoint performance [15,16]. No prior review has integrated FST testing methodology, the full breadth of its disease applications, and its comparative performance as a functional endpoint in interventional trials. This review addresses that gap. It provides an updated overview of current FST protocols with a critical appraisal of factors influencing variability across studies. It then summarizes disease-specific applications, including LCA, RP, choroideremia, Stargardt disease, age-related macular degeneration, birdshot chorioretinopathy, and Jalili syndrome. Finally, it synthesizes trial evidence, along with key limitations of FST, to guide its application as a functional outcome measure.

## 2. Clinical Protocols

### 2.1. Setup

According to the International Society for Clinical Electrophysiology of Vision (ISCEV) and the Imaging and Perimetry Society (IPS), pupil dilation is required for standardized retinal illumination. Proper occlusion of the contralateral eye is also necessary to avoid unintended light stimulation during testing [17].

### 2.2. Dark Adaptation

FST can be performed under light- or dark-adapted conditions. While reported dark adaptation durations vary from none to two hours, a 45 min dark adaptation period is generally recommended [17]. IRDs often require extended dark adaptation, adding complexity to protocol standardization [18,19,20]. Insufficient or inconsistent dark adaptation is a commonly under-reported soure of inter-study variability, which complicates comparison of thresholds across centers [16].

### 2.3. Thresholding

**Figure 1:**
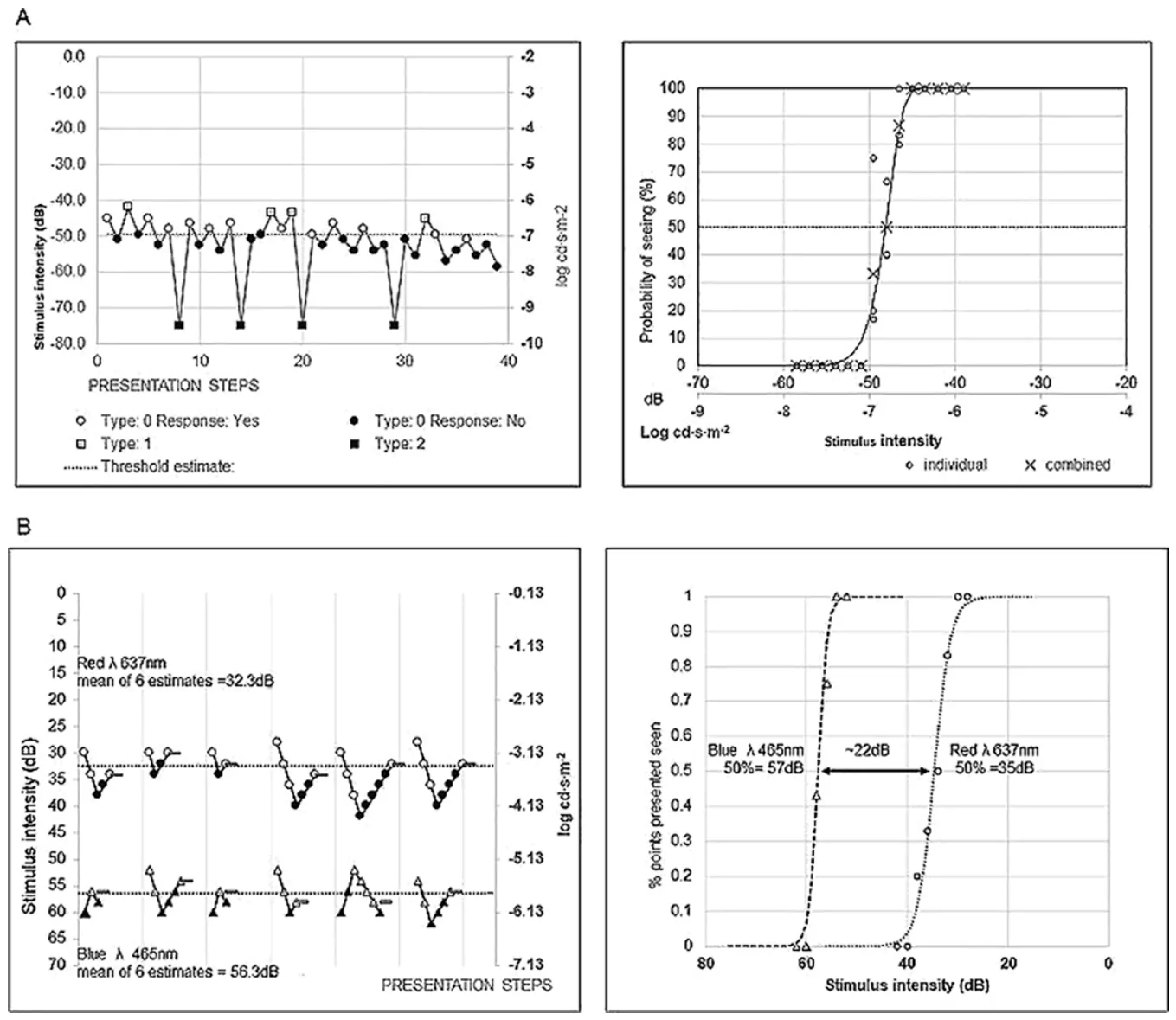
Representative methods for determining full-field stimulus thresholds. (**A**) Example of a psychometric approach in which binary “seen” and “not seen” responses are fit with a modified Weibull function to estimate the stimulus intensity corresponding to a 50% probability of detection. (**B**) Example of a staircase thresholding approach using repeated blue (465 nm) and red (637 nm) stimuli. Multiple staircase runs are averaged to determine threshold sensitivity, and the blue–red threshold difference provides an estimate of relative rod and cone function [17].

Threshold determination is fundamental to FST. The patient responds to incremental light intensities from dim to bright as being ‘seen’ or ‘not seen’, with stimuli presented in achromatic (white) or chromatic (red, green, blue) conditions. Different wavelengths help assess specific photoreceptor functions: blue stimuli predominantly activate rods, red stimuli target L-cones, and green stimuli activate both rods and M-cones [2,21]. The stimulus-response results are modeled using a psychometric function, commonly the two-parameter modified Weibull fit, to estimate the stimulus intensity corresponding to a 50% probability of detection (Figure 1). This psychometric function is generated from the patient’s binary responses across a range of stimulus intensities and provides an objective estimate of retinal sensitivity.

FST methodologies typically involve a single response button, a 200 ms-long stimulus, and a 4-2-2 dB staircase thresholding algorithm. In this approach, the stimulus intensity is adjusted according to the patient’s responses until the threshold is reached, and repeated staircase measurements are averaged to improve reliability (Figure 1). The staircase algorithm is the favored approach because it concentrates stimulus presentations near the patient’s threshold, shortening test duration and reducing patient fatigue. This is an important consideration in children and patients with severe visual impairment who tire quickly. Larger initial step sizes allow rapid convergence toward the approximate threshold, and the smaller terminal steps refine the estimate. An alternative strategy is a full psychometric (method-of-constant-stimuli) approach, which fits the complete stimulus-response curve and yields both threshold and slope. While this approach may be more precise, it requires more presentations and could be more sensitive to response errors. Adaptive Bayesian strategies (ZEST, QUEST/QUEST+, Psi, MLE) have also been proposed to further improve efficiency. No single algorithm has yet been formally validated as superior across diseases, which contributes to methodological heterogeneity between studies [22,23].

Chromatic FST assumes that each wavelength isolates a single photoreceptor class, but this isolation is incomplete. The dominant mediator is not measured directly. Instead, it is inferred from the blue-red threshold difference, where a difference of approximately 25 dB indicates rod mediation and comparable blue and red thresholds indicate cone mediation [24]. However, this inference is unreliable in advanced disease because rod and cone spectral sensitivities can overlap, and the adaptational state determines which mechanism detects the stimulus [25]. Additionally, significant rod loss can shift a rod-mediated blue response toward cone mediation. For these reasons, chromatic FST should be interpreted along with the adaptation state and overall threshold level.

### 2.4. Instrument and Protocol Variability

Because FST is not a single standardized test run on a single instrument, heterogeneity presents as a major barrier to comparing thresholds across studies. A scoping review of 85 sources found that core parameters, including flash luminance equivalent to 9 dB, stimulus color, stimulus duration, and dark adaptation time, are inconsistently applied and under-reported [16]. The Diagnosys Espion system (Diagnosys LLC, Lowell, MA, USA) underlies the commercial D-FST protocol, which uses brief LED flashes and was validated against the earlier FST2 protocol [26]. The Metrovision MonCvONE perimeter (Metrovision, Pérenchies, France), also a widely used FST system, operates with its own stimulus duration and response configuration. Because both platforms allow extensive customization, two centers using the same instrument can run significantly different tests.

The most consequential difference is the luminance assigned to the 0 dB reference, since decibel thresholds are only interpretable relative to this value. In one study, a FST2 protocol was employed that set 0 dB at 0.74 cd·s·m^−2^ with a fixed 200 ms stimulus and a range of roughly +65 yo −25 dB, whereas a D-FST protocol was also applied that set 0 dB at 1.0 cd·s·m^−2^ with a variable stimulus of less than 4 ms and a range of roughly −75 to +15 dB [13]. This illustrates that, even within a single study, thresholds are not interchangeable without conversion. Differing dark adaptation times compound these issues. For example, one study in eyes with CRB1-associated retinal dystrophy used a dark adaptation time of 20 min [27], while many others used the standard time of 45 min [1]. Inconsistent adaptation is also a common but frequently unreported contributor to between-center differences. Finally, chromatic settings add a further layer because the wavelengths used to target rods and cones are not standardized across studies.

These differences carry directly into reliability. In patients with RP and poor acuity tested on a Diagnosys system, coefficients of repeatability were ±0.38 log units for blue, ±0.30 for red, and ±0.59 for white stimuli, with greater variability in mixed-mediation eyes and in older patients [28]. The larger value in white stimuli shows how stimulus color along changes the smallest differnce a protocol can reliably detect. Taken together, differences in 0 dB reference, stimulus duration, chromatic settings, and thresholding algorithms, as discussed previously, make pooling and comparing results across studies difficult.

### 2.5. Patient Education and Repeat Testing

Thorough patient instruction significantly improves test reliability [15]. Patients must understand that the test measures light detection rather than shape or color perception. As with other psychophysical tests, a learning curve is expected. Therefore, multiple assessments (typically at least three) are recommended to obtain a reliable average threshold [17].

## 3. Clinical Applications of FST

### 3.1. Leber Congenital Amaurosis

Leber congenital amaurosis (LCA) encompasses a genetically heterogeneous group of severe retinal dystrophies from birth or early infancy. The disorder predominantly follows an autosomal recessive inheritance pattern, though rare autosomal dominant cases have been reported. Clinically, LCA manifests with profound visual loss, nystagmus, sluggish pupillary responses, and early-onset retinal degeneration, frequently accompanied by hyperopia and Franceschetti’s oculo-digital sign [29].

At the molecular level, LCA is associated with pathogenic variants in over 25 genes, each encoding proteins vital for photoreceptor development, phototransduction, ciliary function, or the retinoid cycle. Key genes frequently implicated include *GUCY2D*, *CEP290*, and *RPE65* [30]. *GUCY2D* plays a crucial role in the phototransduction cascade by facilitating cGMP synthesis. Pathogenic variants in this gene result in persistent photoreceptor hyperpolarization and impaired visual signal transmission. Notably, pathogenic *GUCY2D* variants account for 10–20% of LCA cases, particularly in those with extinguished ERG responses [31]. *RPE65*, encoding retinal pigment epithelium-specific 65 kDa protein, is essential for the visual cycle, specifically in regenerating 11-cis-retinal. Loss-of-function pathogenic variants disrupt chromophore recycling, leading to retinal dysfunction. Importantly, RPE65-associated LCA has been successfully treated with gene augmentation therapy using voretigene neparvovec-rzyl (VN, Luxturna^®^, Spark Therapeutics, Philadelphia, PA, USA), an adeno-associated virus (AAV2)-based treatment administered as a subretinal injection [11]. *CEP290*, a ciliary transition zone protein, is vital for maintaining the integrity of photoreceptor outer segments. Pathogenic variants disrupt ciliary trafficking, leading to severe, early-onset photoreceptor degeneration. These variants are often associated with LCA10, the most common subtype of LCA, and are a major focus of emerging antisense oligonucleotide-based therapies [32].

Optical coherence tomography (OCT), fundus imaging, and fluorescein angiography (FA) are commonly used for diagnosing and monitoring LCA. However, these imaging modalities exhibit considerable inter-subject variability and have inherent limitations in assessing disease progression and treatment response in LCA. OCT often reveals outer retinal atrophy, ellipsoid zone loss, and varying degrees of retinal lamination preservation, findings that correlate with specific genetic subtypes of LCA [33]. Fundus photography may appear normal or show pigmentary changes, vascular attenuation, or macular atrophy, while FA typically demonstrates normal or attenuated choroidal fluorescence, depending on the disease stage and subtype [34]. Despite providing valuable structural insights, these assessments of morphologic changes offer only a limited reflection of functional vision, as retinal remodeling and neuronal plasticity can occur independently of gross anatomical changes. Given that LCA patients often exhibit minimal or non-recordable ERG responses due to widespread rod and cone dysfunction, functional assays such as full-field stimulus testing (FST) have become essential for evaluating residual visual function. FST has emerged as a key outcome measure in clinical trials to assess treatment efficacy [1,35].

Some of the earliest utilizations of FST were in clinical trials of VN, a gene therapy for RPE65-associated inherited retinal diseases (IRDs). Phase I trials demonstrated short-term improvements in FST thresholds following treatment in one study, while another study reported sustained efficacy and stability over three years, leading to subsequent clinical trials [36,37]. Furthermore, phase III trials provided stronger evidence of long-term improvements in light sensitivity, showing FST threshold reductions of 2–3 log units in treated patients compared to controls. Across all treatment groups, FST performance improved by more than 1.8 log_10_ units (log_10_ [cd·s/m^2^]) by Day 30 following vector administration. By Year 1, the gain reached 2.3 log_10_ units and remained stable for up to 4 years [38]. Another study demonstrated the relationship between full-field stimulus threshold (FST) and Goldmann visual field (GVF) improvements after voretigene neparvovec treatment. The study showed that as FST sensitivity improves, GVF area also expands, indicating a parallel trend in functional vision enhancement [39]. These findings suggest that FST can serve as a quantitative measure correlating with clinically meaningful visual field improvements. These improvements correlated with better performance in multi-luminance mobility testing [11,12,38]. One study observed FST enhancements across all tested wavelengths (white, blue, and red light), indicating clinically meaningful gains in visual function [11]. Numerous real-world studies have continued to use FST to monitor LCA patients and assess VN’s effectiveness. Two studies reported significant FST reductions of 2–4 log units, particularly in response to blue light stimuli, aligning with earlier clinical trial findings that demonstrated enhanced rod photoreceptor function [39,40]. Additionally, even in cases where perifoveal chorioretinal atrophy developed post-treatment, FST gains persisted, suggesting that such atrophy may not negate broader improvements in visual function achieved through therapy [41].

Building on its success in RPE65-associated LCA, FST has also been assessed in LCA cases caused by pathogenic *CEP290* and *GUCY2D* variants, demonstrating its potential as a viable endpoint for future clinical trials [14,42]. In a natural history study of *CEP290*-associated IRDs, FST was used to monitor disease progression over 12 months, detecting subtle changes in retinal function even when other clinical measures remained stable [14]. FST has already been incorporated as an outcome measure in phase 1/2 clinical trials for *CEP290* and *GUCY2D*, once again supporting its use as a sensitive tool for detecting treatment-related improvements in light sensitivity. For pathogenic *GUCY2D* variants, clinical trials include ATSN-101, an AAV-mediated gene augmentation therapy designed to deliver a functional copy of *GUCY2D* [13]. For pathogenic *CEP290* variants, investigational therapies include EDIT-101, a CRISPR-Cas9 gene-editing approach targeting the deep-intronic mutation responsible for aberrant splicing, and sepofarsen, an antisense oligonucleotide therapy that also targets this mutation [43,44].

Although these studies consistently support FST as a functional endpoint, they differ greatly in design, population, and follow-up, all of which can impact direct comparison of reported FST outcomes (Table 1). The trials span therapeutic classes from AAV gene augmentation for *RPE65* and *GUCY2D* to gene editing and antisense oligonucleotides for *CEP290*. Additionally, many of the sample sizes are small as a result of the rarity of LCA. Only the VN program reached randomized phase III evaluation, while the others are open-label, uncontrolled, or observational. Follow-up ranges from short-term phase I reporting to durability data extending to 3–4 years for VN. The magnitude of FST improvement was also dependent on genotype and the intervention, with therapies for *RPE65* and *GUCY2D* producing large rod-mediated gains of 2–3 log units. These design and population differences, rather than differences in the reliability of FST itself, likely account for much of the variability in reported effect sizes.

FST does have some important limitations in LCA. Because it depends on a subjective behavioral response, it is subject to a learning effect and to attention and comprehension difficulties, which are particularly relevant in the young children who make up much of the LCA population. Additionally, test–retest variability sets a floor on how small a change can be interpreted as real. In advanced disease such as LCA, thresholds can reach the upper stimulus limit and may not be measurable in the most severely affected patients.

### 3.2. Retinitis Pigmentosa

Retinitis pigmentosa (RP) is a genetically heterogeneous group of inherited retinal dystrophies characterized by progressive photoreceptor degeneration, ultimately leading to visual impairment and blindness. In the early stages, the disorder primarily affects rod photoreceptors, resulting in nyctalopia and peripheral visual field constriction. As the disease progresses, cone photoreceptors become involved, leading to central vision loss and diminished color perception. RP affects approximately 1 in 3000 to 1 in 5000 individuals worldwide and follows autosomal dominant, autosomal recessive, and X-linked inheritance patterns, with rare cases of digenic and mitochondrial inheritance also reported [7,46].

More than 80 genes have been identified in retinitis pigmentosa (RP), encoding proteins crucial for phototransduction, ciliary transport, and photoreceptor maintenance. The molecular pathology of RP ultimately leads to photoreceptor apoptosis through multiple interconnected mechanisms. In RHO-related autosomal dominant RP, protein misfolding triggers the unfolded protein response and endoplasmic reticulum (ER) stress, activating pro-apoptotic pathways via CCAAT/enhancer-binding protein homologous protein (CHOP) and caspase signaling [47]. Disruptions in the phototransduction cascade, such as those caused by pathogenic *PDE6B* variants, result in aberrant cyclic GMP accumulation, sustained depolarization, and dysregulated calcium homeostasis, leading to photoreceptor degeneration through calpain activation and mitochondrial dysfunction [48]. Oxidative stress further exacerbates retinal degeneration as excessive reactive oxygen species accumulate due to mitochondrial dysfunction and diminished antioxidant capacity in degenerating photoreceptors. This contributes to lipid peroxidation, DNA damage, and activation of apoptosis-inducing factor [49]. Ciliary dysfunction also plays a crucial role in RP pathogenesis, as proteins encoded by *RPGR*, *RPGRIP1L*, and *CEP290* regulate intracellular trafficking within the photoreceptor connecting cilium. Pathogenic variants in these genes disrupt the transport of key phototransduction proteins, leading to progressive photoreceptor dysfunction and degeneration [50]. Although cone photoreceptors are initially spared, they undergo secondary degeneration due to the loss of metabolic and trophic support from rods. The depletion of rod-derived neurotrophic factors, combined with metabolic starvation and oxidative damage, contributes to gradual cone attrition, ultimately resulting in central vision loss [51].

The clinical presentation of RP is highly variable because of its genetic and phenotypic heterogeneity. However, fundus examination often reveals characteristic retinal findings, including retinal vessel attenuation, bone spicule pigmentation, and a pale optic disc. OCT provides a detailed assessment of structural changes, such as photoreceptor loss, retinal thinning, or cystoid macular edema [52]. Visual field testing, another essential diagnostic tool, typically shows peripheral vision loss in the form of constricted visual fields or ring scotomas. These defects correlate with the extent of rod photoreceptor degeneration and help track disease progression, with only a small central visual field remaining in advanced RP [53]. For assessing retinal function, full-field electroretinography (ffERG) is regarded as the gold standard. In early RP, ffERG demonstrates diminished or absent rod responses, with cone dysfunction becoming evident as the disease advances [7]. While ffERG is useful for tracking early disease progression, rod ERG amplitudes are often undetectable in advanced RP, making it challenging to find suitable functional outcome measures for clinical trials [26]. Since most patients enrolled in clinical trials have advanced RP, identifying an appropriate outcome measure remains a significant challenge. FST has emerged as a potential secondary outcome measure in RP trials, as it has shown strong correlations with both anatomical assessments, such as OCT [10] and patient-reported visual function measured by the Michigan Retinal Degeneration Questionnaire (MRDQ) [24].

**Figure 2:**
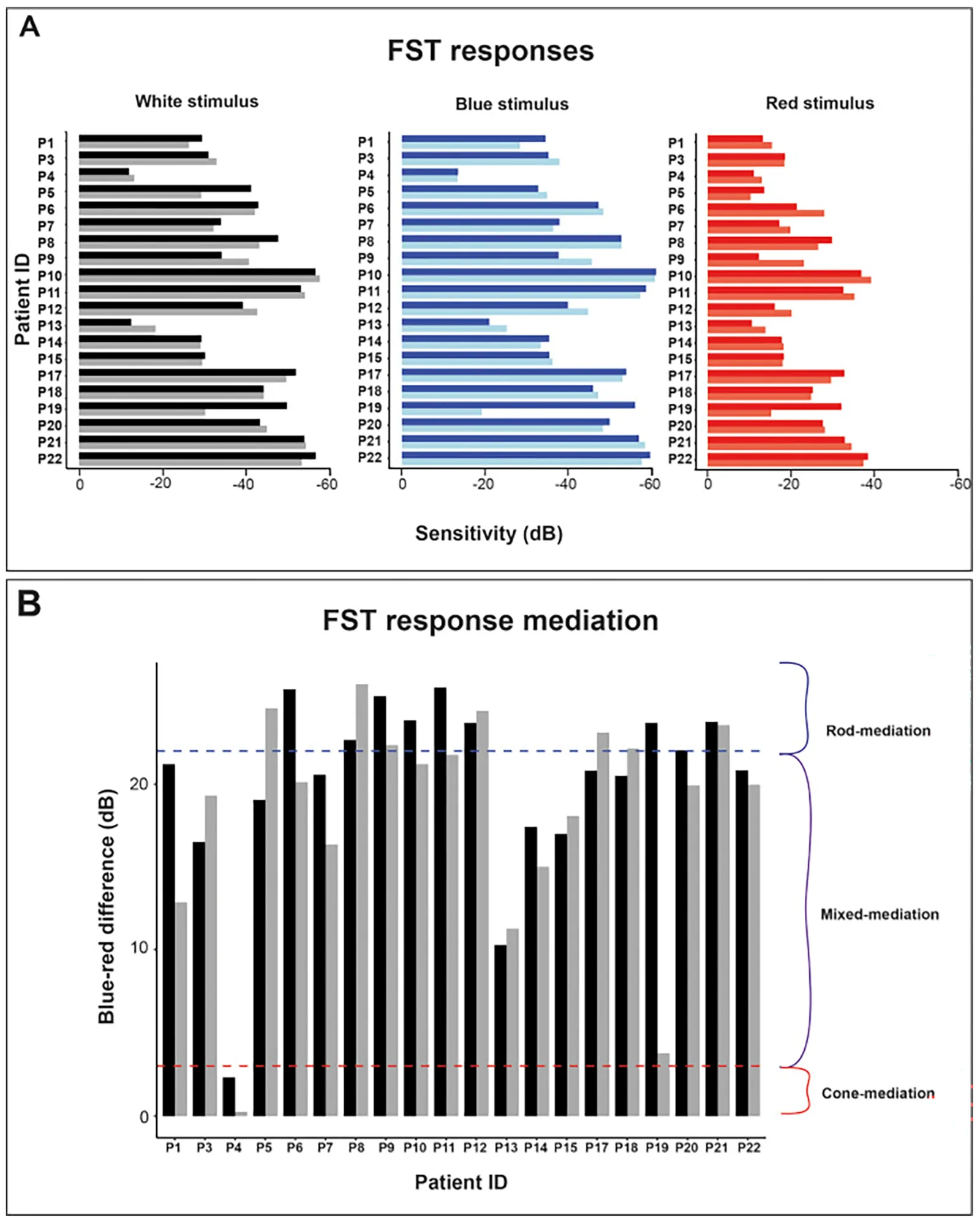
Representative FST results from patients with CRB1-associated retinal dystrophy. (**A**) Threshold sensitivities obtained using white, blue, and red full-field stimuli for each eye. (**B**) Blue-red threshold differences used to classify responses as predominantly cone-mediated, mixed rod- and cone-mediated, or rod-mediated [54].

To date, FST has been frequently utilized in studies characterizing the clinical phenotypes of various pathogenic variants associated with RP (*CRB1*, *PRPF31*, and *PDE6B*) and has been proposed as a valuable endpoint for future therapeutic trials targeting these variants [54,55,56]. Representative FST results from patients with *CRB1*-associated retinal dystrophy are shown in Figure 2. This figure shows how threshold responses to achromatic and chromatic stimuli, combined with the blue-red threshold difference, can be used to distinguish rod-, cone-, and mixed-mediated retinal function. Across these RP studies of various genes, FST measurements remained fairly stable over time, further supporting its reliability as an endpoint. In the Rate of Progression in Usher syndrome type 2 (USH2A)-related Retinal Degeneration (RUSH2A) multicenter study, baseline FST metrics showed a strong association with duration of disease [57]. Furthermore, FST thresholds showed a significant correlation with dark-adapted visual field sensitivity, suggesting their potential as an indicator of rod function in USH2A-related retinal degeneration [58], although it has yet to be determined whether FST is a sensitive measure of disease progression in USH2A. FST has also been used as an outcome measure in a phase I clinical trial for the pathogenic *MERTK* variant, where subretinal injection of rAAV2-VMD2-hMERTK resulted in relatively stable threshold sensitivities over a two-year period [59]. More recently, FST has also been used to assess visual function following a case study of stem-cell-derived retinal organoid transplantation in RP [60].

### 3.3. Choroideremia

Choroideremia (CHM) is an X-linked recessive chorioretinal dystrophy characterized by progressive degeneration of the retinal pigment epithelium (RPE), photoreceptors, and choroid. The estimated prevalence is approximately 1 in 50,000 individuals, with affected males typically experiencing progressive vision loss, while female carriers may exhibit mild or asymptomatic fundus changes due to random X-chromosome inactivation [61,62]. Choroideremia is caused by loss-of-function mutations in the *CHM* gene, which encodes Rab escort protein 1 (REP1). Approximately 30% of pathogenic *CHM* variants are nonsense mutations, leading to premature termination codons and defective REP1 production [63]. REP1 is crucial for Rab GTPase prenylation, a process essential for intracellular vesicular trafficking. Despite its expression in multiple tissues, the retina and choroid are particularly vulnerable due to the high metabolic demands of photoreceptors and RPE cells [64]. The *RPE* relies on Rab-mediated trafficking for essential functions, including photoreceptor outer segment phagocytosis (via Rab27a), intracellular lipid and protein recycling, and vascular support from the choroidal endothelium. *CHM* mutations disrupt these pathways, leading to the accumulation of phototoxic waste from defective outer segment recycling, oxidative stress and impaired lysosomal degradation, and progressive apoptosis of RPE and photoreceptors, ultimately causing retinal and choroidal atrophy. These pathological changes manifest as retinal transparency and increased visibility of scleral vessels [65,66].

Clinically, choroideremia manifests as progressive nyctalopia (night blindness) beginning in early childhood, followed by gradual peripheral visual field constriction and eventual central vision loss in later decades. OCT reveals progressive thinning of the outer retina, loss of the ellipsoid zone, and chorioretinal atrophy. Fundus autofluorescence (FAF) initially shows a characteristic speckled pattern, which gradually transitions to widespread hypoautofluorescence as the disease advances [67,68].

The success of VN in treating LCA caused by pathogenic *RPE65* variants has driven significant research into gene therapies for other IRDs, including choroideremia. Gene therapy approaches include AAV vectors administered by subretinal or intravitreal injection, as well as nonsense suppression therapy and antisense oligonucleotides [69,70,71]. Although no therapies under investigation have yet received FDA approval, defining appropriate outcome measures remains critical as clinical trials advance. A study using retrospective dominance analysis ranked visual acuity (VA) as the least informative among various test modalities for choroideremia clinical trials, suggesting that other visual function assessments may provide more meaningful insights [5]. The decline in FST sensitivity was exponentially associated with a decrease in the area of residual peri- and parafoveal RPE, and FST responses demonstrated a high correlation with both mean and highest macular microperimetry thresholds [72], which supports the use of FST as a complementary tool to guide patient selection and expand eligibility criteria for current and future CHM clinical trials.

### 3.4. Stargardt Disease

While natural history studies and clinical trials have primarily focused on LCA and RP, FST has also been utilized in other IRDs. Stargardt disease, the most common form of juvenile macular dystrophy, is an autosomal recessive disorder with an estimated prevalence of 1 in 8000 to 10,000 individuals. First described by Karl Stargardt in 1909, the disease is primarily caused by pathogenic variants in the *ABCA4* gene, which encodes an ATP-binding cassette transporter responsible for clearing toxic retinoid byproducts from photoreceptor cells [73,74]. Pathogenic variants in *ABCA4* lead to the accumulation of lipofuscin within the RPE, resulting in progressive photoreceptor degeneration and macular atrophy. In rare cases, dominant forms of Stargardt disease (STGD3) are associated with pathogenic variants in *ELOVL4* or *PROM1*, which disrupt photoreceptor outer segment integrity and lipid metabolism [75,76].

Stargardt disease is clinically characterized by progressive bilateral central vision loss, typically presenting in childhood or adolescence, although late-onset forms also occur. Fundoscopic examination commonly reveals a characteristic macular atrophy with a ‘beaten-bronze’ appearance, often accompanied by yellow-white pisciform flecks at the level of the RPE. As the disease progresses, these flecks may extend beyond the posterior pole, and macular atrophy worsens, leading to significant central vision impairment, while peripheral vision remains relatively preserved [77].

Diagnostic evaluation includes multimodal imaging to assess retinal structure and function. FAF reveals hyperautofluorescent flecks corresponding to lipofuscin accumulation, followed by hypoautofluorescent atrophic areas in advanced disease. OCT demonstrates photoreceptor and RPE loss in the macular region. Visual field testing typically reveals central scotomas with relative sparing of the periphery in early disease, although progressive cases may show larger central defects or paracentral involvement [78,79]. ffERG may show normal or mildly reduced responses in early stages but can exhibit more widespread retinal dysfunction in later stages [80]. FST complements mfERG and ffERG in the diagnosis and monitoring of Stargardt disease associated with pathogenic *CNNM4* variants, as rod and cone threshold elevations in affected patients correlate well with OCT and ffERG measurements [25]. While mfERG primarily evaluates macular (cone) function, FST is more sensitive to early rod dysfunction, which is important in the early stages of the disease. By incorporating FST alongside mfERG and ffERG, a more comprehensive understanding of both cone and rod dysfunction can be achieved, improving diagnosis and monitoring in Stargardt disease.

### 3.5. Age-Related Macular Degeneration

Age-related macular degeneration (AMD) is a progressive retinal disorder and the leading cause of irreversible central vision loss in individuals over 50 years of age. It is classified into two major forms: dry (non-neovascular, atrophic) AMD, which accounts for approximately 85–90% of cases, and wet (neovascular, exudative) AMD, which is responsible for most severe vision loss associated with the disease [81,82].

The pathophysiology of AMD is complex and multifactorial, involving various factors such as genetic predisposition, oxidative stress, inflammation, and dysregulation of lipid metabolism. Variants in complement factor genes (*CFH*, *CFI*, *C3*, *C2*/*CFB*) and lipid transport genes (*APOE*, *ABCA1*, *LIPC*) have been associated with disease susceptibility, contributing to chronic RPE dysfunction and progressive photoreceptor degeneration [83,84].

Dry AMD is characterized by the accumulation of drusen, extracellular, lipid-rich deposits at the level of Bruch’s membrane, along with RPE changes such as pigmentary abnormalities and, in advanced stages, geographic atrophy. In contrast, wet AMD is marked by choroidal neovascularization (CNV), leading to subretinal fluid, hemorrhages, exudates, and eventual fibrosis, resulting in rapid and severe central vision loss [85,86].

OCT is considered the gold standard for diagnosing and monitoring AMD, providing high-resolution cross-sectional imaging of drusen, subretinal fluid, RPE atrophy, and CNV membranes. OCT is particularly sensitive in detecting early changes, including retinal fluid, that may not be visible on FA [87]. Although FA remains valuable for confirming the presence of CNV and assessing leakage patterns, its role in ongoing monitoring is increasingly supplanted by OCT. The combination of OCT with indocyanine green angiography (ICGA) further enhances the assessment of CNV, offering detailed insights into the morphology and activity of the neovascular network, which aids in better management of wet AMD [88]. Anti-vascular endothelial growth factor (VEGF) injections have transformed the treatment landscape for wet AMD over the past two decades. VA and OCT are commonly used as endpoints in anti-VEGF clinical trials and routine clinical practice to guide treatment decisions, with OCT being particularly valuable for assessing retinal fluid and disease activity. Recently, FST has been proposed as a potential independent measure of visual function in wet AMD. A study on treatment-naïve wet AMD eyes found a correlation between central foveal thickness and blue FST retinal sensitivity after two anti-VEGF injections, which persisted following a third injection. Notably, no significant correlation was observed between central foveal thickness and VA beyond baseline, suggesting that FST may serve as a more sensitive indicator of disease activity [89].

These findings support FST as an additional outcome measure in AMD and other maculopathies. With the FDA approval of two complement inhibitor therapies for geographic atrophy, FST may also serve as a valuable tool for monitoring patients in clinical practice and assessing treatment effects in future geographic atrophy trials [90].

### 3.6. Birdshot Chorioretinopathy

Birdshot chorioretinopathy (BSCR) is a rare, bilateral posterior uveitis characterized by chronic inflammation affecting both the choroid and retina [91]. It primarily occurs in middle-aged individuals of European descent and has a strong genetic association with the HLA-A29 allele. Patients with BSCR often experience a range of visual disturbances, including decreased visual acuity, floaters, nyctalopia (difficulty seeing in low-light conditions), dyschromatopsia (altered color perception), photopsia (flashes of light), and increased sensitivity to glare. These symptoms typically develop gradually and affect both eyes simultaneously.

The pathogenesis of BSCR is not yet fully understood, but evidence suggests an autoimmune mechanism in which the body’s immune system mistakenly targets retinal and choroidal tissues. The strong HLA-A29 association indicates a role in antigen presentation, and recent studies have implicated endoplasmic reticulum aminopeptidase 2 (ERAP2) in disease development. Pathogenic variants in ERAP2 may alter antigen processing, leading to an aberrant immune response against ocular structures. Additionally, histopathological findings reveal lymphocytic infiltration in the choroid, prelaminar optic nerve head, and retinal blood vessels, emphasizing the inflammatory nature of BSCR. Understanding these mechanisms is crucial for developing targeted therapies and managing the progressive vision loss associated with the disease.

Multimodal imaging techniques, including enhanced depth imaging optical coherence tomography (EDI-OCT), OCT angiography, FA, ICGA, ffERG, VA assessment, and visual field testing, are essential for diagnosing BSCR [92,93,94,95,96,97,98,99]. Additionally, FST has proven valuable in BSCR diagnosis, as it provides an objective measure of visual function even in individuals with severe visual impairment. Studies have demonstrated its utility in assessing retinal dysfunction in BSCR, with findings showing that changes in choroidal thickness over time correlate with disease progression [91].

### 3.7. Jalili Syndrome

Jalili syndrome is a rare autosomal recessive disorder characterized by the coexistence of cone-rod dystrophy and amelogenesis imperfecta (AI), impacting both vision and dental health. Affected individuals experience progressive visual impairment that may ultimately progress to no light perception. This deterioration presents as reduced visual acuity, photophobia, and nystagmus. Fundoscopic examinations often reveal retinal pigment epithelium atrophy, vascular attenuation, and macular changes. ffERG typically shows reduced or extinguished cone responses with progressive rod dysfunction. In addition to ocular symptoms, patients exhibit dental anomalies consistent with AI, including hypoplastic or hypomineralized enamel, leading to discolored and fragile teeth prone to damage [100,101,102,103,104,105].

The syndrome is caused by biallelic pathogenic variants in the *CNNM4* gene, which encodes a putative metal transporter involved in magnesium homeostasis. Pathogenic variants in *CNNM4* disrupt normal magnesium transport, adversely affecting the development and function of retinal cells and ameloblasts, the cells responsible for enamel formation. This disruption leads to the clinical manifestations observed in Jalili syndrome [106,107]. Recent studies have provided deeper insights into the pathogenicity of *CNNM4* variants. Functional analyses indicate that certain pathogenic variants may lead to decreased mRNA and/or protein stability, rather than mislocalization, thereby impairing the protein’s function. These findings underscore the critical role of *CNNM4*-mediated magnesium transport in the pathogenesis of Jalili syndrome [108].

Diagnosis of Jalili syndrome involves a combination of clinical evaluation, ophthalmic assessments, dental examination, and genetic testing. Clinically, patients present with progressive visual loss, photophobia, and enamel defects from an early age. Ophthalmic evaluations, including fundus examination and ffERG, are essential to assess retinal function and structural integrity. Dental assessments reveal characteristic enamel defects affecting both primary and permanent dentition. Confirmatory diagnosis is achieved through genetic testing, identifying pathogenic variants in the *CNNM4* gene [100]. Understanding the clinical features and genetic basis of Jalili syndrome is crucial for accurate diagnosis, patient counseling, and the development of targeted management strategies.

FST has been used to investigate cone pathway dysfunction in Jalili syndrome associated with pathogenic CNNM4 variants [102]. FST provides an objective measure of visual function, particularly in cases where standard clinical tests may be inconclusive due to severe visual impairment. Its application in Jalili syndrome highlights its potential as a valuable tool for assessing retinal dysfunction in inherited retinal diseases.

## 4. Advantages and Limitations of FST

**Figure 3:**
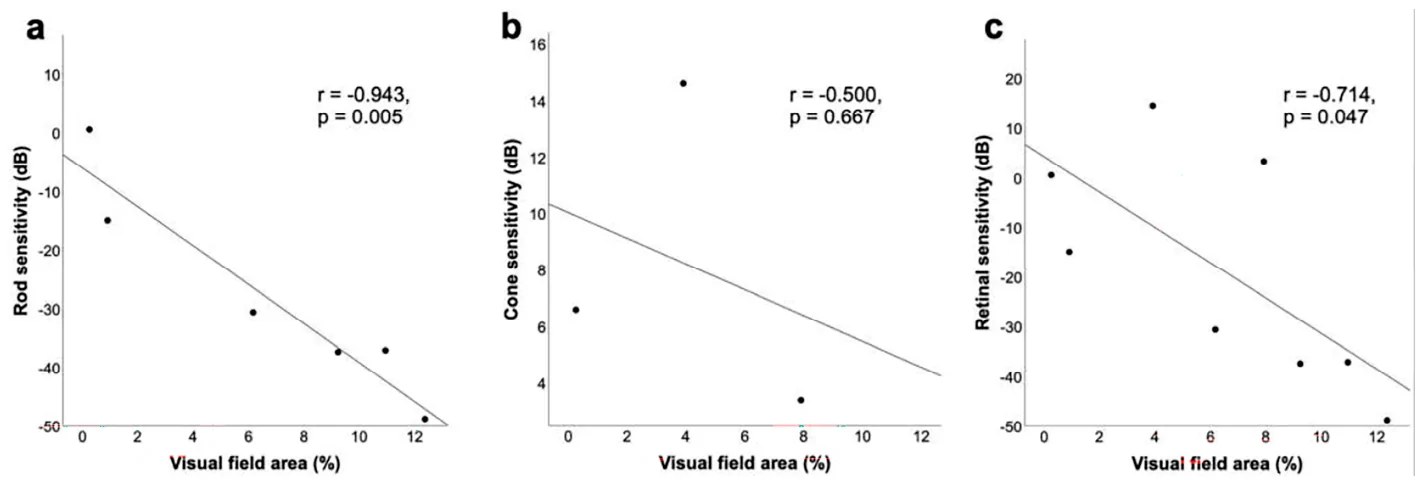
Relationship between residual visual field area and retinal sensitivity measured by FST in patients with advanced RP and only peripheral visual field islands. (**a**–**c**) Correlations between residual visual field area and rod-mediated, cone-mediated, and overall retinal sensitivity, respectively [4].

FST has emerged as a valuable visual function outcome measure, offering a rapid and convenient assessment of retinal sensitivity, which is particularly suitable for both children and older adults. Unlike pattern-based electrophysiological tests, FST does not require precise fixation, making it ideal for patients who have difficulty maintaining steady gaze due to age-related conditions or poor visual acuity. Additionally, FST is relatively simple to perform, requiring only a light perception response, making it well suited for young children who may struggle with more complex visual tasks and for older adults with advanced retinal degeneration. Even in advanced retinal disease with only small islands of residual visual field, FST can still quantify residual retinal sensitivity. As shown in Figure 3, retinal sensitivity measured by FST correlates with residual visual field area, particularly for rod-mediated responses [4]. This supports its utility as a functional outcome measure in patients with severe visual field loss.

However, it has some limitations. First, FST is highly sensitive to external factors such as pupil size and patient attention. To ensure optimal accuracy, testing should be conducted with fully dilated pupils, as conditions like miosis may compromise results. Additionally, since FST relies on subjective responses, patients, particularly younger individuals with LCA, may struggle with attention or test comprehension [15]. Second, data analysis can be challenging in cases of media opacities, severe fixation instability, or profound visual impairment, all of which are common in patients undergoing FST. In advanced retinal degenerations, FST measurements may approach the upper limit of the stimulus dynamic range, potentially limiting the test’s ability to detect residual visual function. However, advancements in testing strategies may help mitigate this limitation.

FST remains in its early stages as an outcome measure, with no established consensus on a standardized protocol for clinical or research use. Studies have demonstrated considerable variability in test parameters, including dark adaptation times, stimulus settings, patient interface, and preparation, all of which can contribute to inconsistencies across testing sites [109]. This variability is partly attributable to the flexibility of commercially available FST systems, which allows for multiple customizations to accommodate individual patients but also introduces ambiguity in the resulting psychometric function. In 2024, ISCEV and IPS published a report aimed at improving consistency in testing methodology and reporting; however, further standardization efforts are still needed [17].

## 5. Future Perspectives

As novel therapeutic approaches for retinal diseases continue to evolve, the clinical importance of FST is expected to increase substantially. Gene augmentation therapy, CRISPR-based gene editing, RNA-based therapeutics, stem cell transplantation, optogenetic therapy, and neuroprotective interventions all require sensitive functional outcome measures capable of detecting changes in patients with severe visual impairment. Because conventional visual acuity, visual field testing, and electroretinography frequently reach floor effects in advanced retinal degeneration, FST provides a unique opportunity to quantify residual retinal function throughout the disease spectrum.

Future efforts should focus on establishing standardized international testing protocols. Although the recent ISCEV and Imaging and Perimetry Society recommendations have improved methodological consistency, further harmonization of dark adaptation time, stimulus characteristics, threshold algorithms, pupil preparation, and reporting standards will facilitate comparisons among clinical studies and multicenter therapeutic trials. Standardization will also improve the reproducibility of FST measurements and strengthen their acceptance by regulatory agencies.

Technological advances are also likely to improve FST performance. Automated threshold estimation algorithms, artificial intelligence-assisted analysis, and adaptive psychophysical testing strategies may reduce test duration while improving measurement precision. Portable testing systems and remote monitoring platforms may eventually permit longitudinal assessment outside specialized clinical centers, thereby expanding access for patients with inherited retinal diseases.

Rather than serving as a standalone measure, FST is increasingly being incorporated into multimodal functional assessment. Combining FST with optical coherence tomography, fundus autofluorescence, microperimetry, full-field electroretinography, dark adaptometry, and patient-reported outcome measures provides complementary information regarding retinal structure and function. Such multimodal approaches are expected to improve patient selection, monitor disease progression more accurately, and provide comprehensive evaluation of treatment efficacy.

As additional retinal therapies enter late-stage clinical development, FST is likely to become an increasingly important functional endpoint in natural history studies, clinical trials, and post-marketing surveillance. Continued validation in larger multicenter cohorts will further establish its role as a standardized measure of visual function for patients with severe retinal disease.

## 6. Conclusions

Full-field stimulus threshold testing has become an important psychophysical method for evaluating global retinal sensitivity, particularly in patients with severe retinal degeneration for whom conventional functional tests are often unreliable or non-recordable. Its fixation-independent design, ability to assess both rod- and cone-mediated visual function, and applicability across a wide range of inherited and acquired retinal diseases have established FST as a valuable tool for clinical evaluation and therapeutic monitoring.

Growing evidence from natural history studies and interventional clinical trials supports the use of FST as a sensitive outcome measure for detecting treatment-related changes following gene therapy, stem cell transplantation, and other emerging retinal interventions. When interpreted together with structural imaging and complementary functional assessments, FST provides a comprehensive evaluation of residual visual function that cannot be obtained using any single modality alone.

Although further standardization of testing protocols and validation across different platforms remain necessary, recent international guidelines represent an important step toward broader clinical implementation. As innovative retinal therapies continue to expand, FST is expected to play an increasingly important role in clinical practice, multicenter therapeutic trials, and regulatory evaluation of novel treatments for retinal diseases.

## Figures and Tables

**Table 1:** A summary of major LCA trials and studies using FST as a functional outcome.

Study	Gene	Intervention	Study Design	Sample (Patients)	Follow-Up	FST Outcome Summary
Hauswirth et al. (2008) [36]	*RPE65*	AAV2 augmentation	open-label, phase I trial	3	≤90 days	Improved thresholds; magnitude not quantitatively reported at cohort level
Jacobson et al. (2012) [45]	*RPE65*	AAV2 augmentation	Open-label, phase I trial	15	≤3 years	1.59 log unit improvement (blue light); sustained/stable to 3 years
Maguire et al. (2019) [38], Maguire et al. (2021) [12]	*RPE65*	Voretigene neparvovec	Open-label, randomized, controlled phase III trial	21	≤4 years	~2.3 log unit improvement at Year 1 (white light); stable through Year 4
Deng et al. (2022) [39]	*RPE65*	Voretigene neparvovec (real-world data)	Retrospective Cohort	14	≤12 months	~2.1 log unit improvement (white light)
Lorenz et al. (2024) [40]	*RPE65*	Voretigene neparvovec (real-world data)	Retrospective Cohort	19	≤32 months	Improved thresholds at all ages (blue light); 8/8 pediatric eyes ≥10 dB (*p* < 0.05)
Jacobson et al. (2017) [42]	*GUCY2D*	Observational (no intervention)	Cross-sectional	28	No follow-up	Baseline data; wide range of rod-mediated dysfunction indicated
Yang et al. (2024) [13]	*GUCY2D*	ATSN-101 (AAV5 augmentation)	Open-label, phase I/II trial	15	≤12 months	20.3 dB (~2 log unit) improvement (white light) at 12 months
Pierce et al. (2024) [14]	*CEP290*	Observational (no intervention)	Natural History	23	≤12 months	Decreased 0.33 log units (blue light) and 0.39 log units (white light) at 12 months
Cideciyan et al. (2022) [43]	*CEP290*	Sepofarsen (antisense oligonucleotide)	Open-label, phase I/II trial	11	≤3 months	1.75 log unit improvement (white light)
Pierce et al. (2024) [44]	*CEP290*	CRISPR-Cas9 editing	Open-label, phase I/II trial	14	≤2 years	0.34 log unit improvement (blue light)

## Data Availability

No new data were created or analyzed in this study. Data sharing is not applicable to this article.
